# Evaluation of A Simple DNA Extraction Method and Its Combination with Loop-Mediated Isothermal Amplification Assays for Rapid *Plasmodium knowlesi* Diagnosis

**DOI:** 10.3390/tropicalmed8080389

**Published:** 2023-07-29

**Authors:** Meng-Yee Lai, Mohd Hafizi Abdul Hamid, Jenarun Jelip, Rose Nani Mudin, Yee-Ling Lau

**Affiliations:** 1 Department of Parasitology, Faculty of Medicine, Universiti Malaya, Kuala Lumpur 50603, Malaysia; mengylai11@yahoo.com; 2 Vector Borne Disease Sector, Ministry of Health, Putrajaya 62000, Malaysia; dr.mhafizi@moh.gov.my (M.H.A.H.); jenarun@moh.gov.my (J.J.); drrose@moh.gov.my (R.N.M.)

**Keywords:** *P. knowlesi*, diagnosis, LAMP, SYTO-9, SYBR green, colorimetric, malaria

## Abstract

The initial and vital stage in the diagnosis of malaria involves extracting DNA. The efficiency of malaria testing is restricted by the multiple steps involved in commercial DNA extraction kits. We attempted to improve an existing loop-mediated isothermal amplification (LAMP) for the detection of *Plasmodium knowlesi* by using a simple DNA extraction approach, making it a feasible option for mass screening. We utilized a simple nucleic acid extraction method directly from whole blood for the detection of *P. knowlesi*, taking only 5 min to complete. The extracted DNA was evaluated by two fluorescent-based LAMP and one colorimetric-based LAMP assay. The detection limit for both SYTO-LAMP and SYBR green-LAMP was 0.00001% and 0.0001% parasitemia, respectively. Meanwhile, neutral red-LAMP had a detection limit of 0.01% parasitemia. Combining this simple and inexpensive DNA extraction method, SYTO-LAMP could serve as an alternative molecular diagnosis for the detection of *P. knowlesi* and other human *Plasmodium* spp.

## 1. Introduction

According to World Malaria Report 2022, there has been a significant rise in the number of humans infected with *P. knowlesi* in Malaysia as well as other South-East Asian regions. For the past four years, there have been no indigenous human malaria cases or deaths reported in Malaysia. However, a total of 17,125 *P. knowlesi* cases and 48 deaths have been reported in Malaysia since 2017 [1]. This parasite was initially thought to infect only monkeys, but it began to infect and adapt to humans since there was a reported case in 2004 [2]. Cases have been reported not only in South-East Asia but also in other areas through travelers [3]. As recommended by the World Health Organization (WHO), both microscopy and rapid diagnostic tests (RDTs) are used as the confirmation diagnosis tool in suspected malaria patients [4]. Microscopic examination remains the gold standard for malaria diagnosis. Under the examination of microscopy, *P. knowlesi* is always misdiagnosed as *P. falciparum* or *P. malariae* due to their similar morphology. Hence, the delay in treatments is happening. Traditional rapid diagnostic kits (RDTs) are frequently used due to their convenience. However, these kits offer poor sensitivity and rarely detect patients with low parasitemia. Of note, some of the RDT kits displayed an unequal sensitivity towards different *Plasmodium* species. In 2013, Maltha et al. reported that RDT sensitivity is good for *P. falciparum*, but only moderate for *P. vivax* (66.0–88.0%) and poor for *P. ovale* (5.5–86.7%) and *P. malariae* (21.4–45.2%) [5]. A previous study by Tang et al. indicated that RDTs had only 36.5 % (*P. o. curtisi*) and 75.0% (*P. o. wallikeri*) [6]. Recently, Wu et al. reported that RDT had a low detection rate (56.5%) among *P. malariae* cases from returned international travelers in China [5]. Overall, the sensitivity of RDTs greatly depends on the parasitemia of the samples [5,6,7]. To date, there is no RDT for the specific detection of *P. knowlesi* samples available on the market. Therefore, a rapid, sensitive, and accurate diagnosis tool is urgently required as *P. knowlesi* has a short and deadly life cycle (24 h).

To eliminate malaria mortality, molecular diagnostics methods, such as nested polymerase chain reaction (PCR), real-time PCR, semi-nested PCR, multiplex PCR, etc., are at the forefront for the detection of *Plasmodium* spp. Among these methods, nested PCR is a widely used nucleic acid amplification technology for malaria detection due to its high sensitivity and specificity [8,9]. However, PCR’s routine use is limited due to its requirement of a thermocycler, experienced personnel, and a longer turnaround time (approximately 6 h). These limitations can be overcome by using isothermal amplification techniques.

Several isothermal amplification approaches have been developed, including loop-mediated amplification (LAMP), recombinase polymerase amplification (RPA), strand displacement amplification (SDA), helicase-dependent amplification (HDA), whole genome amplification (WGA), nucleic acid sequence-based amplification (NASBA), as well as rolling circle amplification (RCA) [10]. All these methods may operate at different temperatures, strand displacement enzymes, end-point detection methods, and reaction times. LAMP is one of the commonly used isothermal technologies because it enables rapid, simple, sensitive, and specific nucleic acid amplification at a constant temperature (60–65 °C). LAMP was first reported by Notomi et al. in 2000 and it is able to amplify both DNA and RNA (with the addition of reverse transcriptase enzyme) targets. This technique is based on the strand displacement activity of *Bst* DNA polymerase with four to six designed primers targeting the specific regions of a gene. LAMP has proven its superiority over PCR as it can amplify a few copies of DNA to an immense amount in less than 60 min without the requirement of special equipment [11]. Due to its simplicity and sensitivity, LAMP has attracted considerable interest and has been explored for the detection of various infectious and parasitic diseases caused by viruses, bacteria, fungi, protozoa, plant pathogens, and worms [12,13,14,15,16,17,18,19]. However, there is a drawback to LAMP. Since multiple primers are involved in a LAMP assay, non-specific binding may occur due to primer-dimer formation resulting in template-free amplification and, subsequently, leading to false positive results [20]. LAMP amplicons can be evaluated by turbidimetric, agarose gel electrophoresis, lateral flow dipsticks, fluorescent-based, and colorimetric-based methods [21]. The turbidity of the end products is due to the accumulation of magnesium pyrophosphate by-products during the DNA amplification [22]. The turbidity result readout could be visualized by the naked eye or with the use of a turbidimeter. However, relying on turbidity readout as the final LAMP result has been shown to be less reliable and subjective as compared to colorimetric-based and fluorescent-based LAMP assays. In the work presented here, we use fluorescent-based (SYTO-9 and SYBR green) and colorimetric-based (neutral red) LAMP assays to amplify *P. knowlesi* extracted through a simple extraction method. To the best of our knowledge, this is the initial endeavor to employ neutral red in LAMP as a diagnostic technique for malaria.

Nowadays, the most used DNA preparation methods in the laboratory are through commercial DNA extraction kits. Researchers and laboratory technologists are aware that the kits are costly and involve multiple steps of DNA extraction and purification protocols. Involving numerous processing steps can be attributed to the risk of contamination and cost per reaction. Entire protocols may take approximately 50 min from sample to PCR-ready DNA. Thus, simple, cost-effective, and rapid DNA extraction methods are highly demanded. 

Several rapid DNA extraction methods from malaria-dried blood spots have been described. Many of these have great diagnostic performance. Xiang et al. employed a simple alkali lysis method to extract *P. falciparum* DNA from filter paper blood samples. This group of researchers managed to detect down to 0.01% parasitemia [23]. In 2005, Bereczky et al. described a Tris-EDTA (TE) buffer-based method for the extraction of *P. falciparum* DNA from blood dried on filter paper. The sensitivity of detection relied on the types of filter paper. They obtained 100% sensitivity by using 3 MM Whatman filter paper [24]. In 2013, Migue et al. reported a Chelex-saponin method of extraction of *P. falciparum* and *P. vivax* blood spots and obtained approximately 66% and 31% sensitivity for *P. falciparum* and *P. vivax*, respectively [25].

Presently, there are not many published reports on rapid DNA extraction methods from malaria whole blood. In a previous study by Modak et al. [26], a basic DNA extraction technique was devised employing phosphate-buffered saline (PBS) on whole blood and saliva specimens, enabling the identification of falciparum and vivax malaria via LAMP. However, the number of samples evaluated was restricted to those provided by the National Institute of Allergy and Infectious Diseases (NIAID). Our investigation involved adapting this approach to extract DNA and diagnose knowlesi malaria from clinical samples. Furthermore, in the previous study, the test’s analytical sensitivity was determined through DNA concentration estimation, but in our research, we conducted a parasite culture to precisely establish the detection limit.

In this study, we report and compare the performance of the SYTO-LAMP, SYBR green-LAMP, and neutral red-LAMP assays to diagnose malaria samples extracted using a simple method.

## 2. Materials and Methods

### 2.1. Source of Samples

A total of 97 blood samples (*P. knowlesi*, n = 67, non-*P. knowlesi*, n = 10, and healthy donors, n = 20) were involved in this study. Non-*P. knowlesi* samples were 5 *P. falciparum*, 3 *P. vivax*, 1 *P. malariae*, and 1 *P. ovale*). These samples were obtained from University Malaya Medical Center (UMMC), Sarawak State Health Department, and district hospitals in Selangor, Pahang, Kelantan, Melaka, Terengganu, Johor, and Perak. Parasitemia range of these samples was 0.001–8.78%. Ethics approvals were obtained from the Medical Research and Ethics Committee (MREC) of the Ministry of Health Malaysia (NMRR-15-672-23975) and the Medical Ethics Committee of the University Malaya Medical Center (MEC Ref. No. 817.1). These samples were previously confirmed by microscopic examination and nested PCR targeting *18S rRNA* gene. 

The primers used for the detection of *Plasmodium* sp., *P. falciparum*, *P. vivax*, P. malariae, and *P. ovale* have been previously described by Snounou et al. [27]. Meanwhile, the primers used for the detection of *P. knowlesi* were previously described by Imwong et al. [28]. Nested 1 PCR was used for genus-specific confirmation and nested 2 PCR was used for species-specific identification. The reaction mixture for the nested 1 PCR step included 12.3 μL distilled water, 1× Green GoTaq^®^ Flexi buffer, 2 mM magnesium chloride (MgCl_2_), 200 µM deoxyribonucleotide triphosphate (dNTPs), 1 U *Taq* DNA polymerase (Promega Corporation, Madison, WI, USA), 200 nM rPLU1 and rPLU5 each (primer for *Plasmodium* spp.), and 4 μL DNA template. The cycling conditions of nested 1 were as follows: 94 °C for 4 min, 35 cycles of denaturation at 94 °C for 30 s, annealing at 55 °C for 1 min, and elongation at 72 °C for 1 min, and final extension at 72 °C for 10 min. As for nested 2 PCR, 5 sets of species-specific primers were involved, PkF1140/PkR1550, rOVA1WC/rOVA2WC, VIV1/VIV2, FAL1/FAL2, and MAL1/MAL2. The reaction mixture for nested 2 was similar to nested 1 except for the primers used. The cycling conditions for nested 2 were also similar to nested 1 except for the annealing temperature, which was 58 °C. PCR products were run on 1.5% agarose gel and visualized through Gel Doc XR+ gel documentation (Bio-Rad Laboratories Inc., Hercules, CA, USA).

### 2.2. Alternative DNA Extraction from Blood Samples

The preparation of DNA from the infected blood was adapted from Modak et al. with minor modifications [26]. One microliter of infected blood was diluted in 1000 µL 1× PBS. The diluted blood was then heated at 95 °C for 5 min to lyse the blood cells and release DNA. Four µL of the extracted DNA was confirmed by nested PCR prior to SYTO-LAMP, SYBR green-LAMP, and neutral red-LAMP.

### 2.3. SYTO-LAMP Assay

SYTO-LAMP reaction was performed by using the *P. knowlesi* primers (Table 1) that have been reported previously [29]. A working stock of 50 µM SYTO-9 green fluorescent nucleic acid stain (Invitrogen Corporation, Waltham, MA, USA) was prepared by using distilled water. The 25 μL reaction mixture consisted of 4 µL DNA template, 8.45 µL distilled water, 2.5 µL of 10× isothermal amplification buffer, 1.5 µL of magnesium sulfate (MgSO_4_), 3.5 µL of deoxynucleoside triphosphate (dNTPs) (10 mM), 1 µL of *Bst* 2.0 WarmStart DNA polymerase (New England Biolabs, Ipswich, MA, USA), 40 pmol of FIP and BIP each, 10 pmol of FLP and BLP each, 5 pmol of F3 and B3 each, and 0.5 µM of SYTO-9 green fluorescent nucleic acid stain. DNA amplification was carried out at 65 °C in BioRad CFX96 real-time PCR machine (BioRad Laboratories, Hercules, CA, USA) for ~60 min.

In order to obtain the most suitable concentration of SYTO 9, different concentrations (0.25, 0.5, 1, and 1.5 µM) were tested. The SYTO 9 was diluted from 5 M stock to the desired final dilutions with sterile distilled water. The SYTO-LAMP assays with different concentrations were performed in duplicate and repeated twice.

### 2.4. SYBR Green-LAMP

SYBR green-LAMP assay was performed by using the *P. knowlesi* primers (Table 1) that have been reported previously. A working stock of SYBR Green (1:400) (Invitrogen Corporation, United States) was prepared by using distilled water. The 25 μL reaction mixture consisted of 4 µL DNA template, 12 µL distilled water, 2.5 µL of 10× isothermal amplification buffer, 1.5 µL of MgSO_4_, 3.5 µL of dNTPs (10 mM), 1 µL of *Bst* 2.0 WarmStart DNA polymerase (New England Biolabs, Ipswich, MA, USA), 40 pmol of FIP and BIP each, 10 pmol of FLP and BLP each, 5 pmol of F3 and B3 each, and 0.5 µL of SYBR Green. DNA amplification was carried out at 65 °C in BioRad CFX96 real-time PCR machine (BioRad Laboratories, Hercules, CA, USA) for ~60 min.

In order to obtain the most suitable concentration of SYBR green, different dilutions (1:200, 1:300, 1:400, and 1:500) were tested. The SYBR green was diluted from 10,000× stock to the desired final dilutions with sterile distilled water. The SYBR green-LAMP assays with different concentrations were performed in duplicate and repeated twice.

### 2.5. Neutral Red-LAMP

To use neutral red (Sigma Aldrich, St. Louis, MO, USA) as the LAMP result readout, 10× low pH buffer (100 mM (NH_4_)_2_SO_4_, 500 mM potassium chloride, 20 mM MgSO_4_ and 1% Tween-20) with pH 8.5 was prepared according to the protocols from Jaroenram et al. [30]. The pH was adjusted by using 1M potassium hydroxide. A working stock of 2.5 mM neutral red was prepared. The final concentration of neutral red used in the LAMP assay was 100 µM. The 25 μL reaction mixture consisted of 7.7 µL distilled water, 2.5 µL of 10× low pH buffer, 1.5 µL of MgSO_4_, 3.5 µL of dNTPs (10 mM), 1 µL of *Bst* 2.0 WarmStart DNA polymerase (New England Biolabs, Ipswich, MA, USA), 40 pmol of FIP and BIP each, 10 pmol of FLP and BLP each, 5 pmol of F3 and B3 each, 1 µL of neutral red, and 4 µL of the extracted DNA. DNA amplification was performed at 65 °C in a heating block for ~40 min. The reaction was further deactivated by incubation at 80 °C, for 2 min.

In order to obtain the most suitable concentration of neutral red, different final concentrations (50, 75, 100, and 125 µM) were tested. Neutral red was diluted from 2.5 mM stock to the desired final dilutions with sterile distilled water. The neutral red-LAMP assays with different concentrations were performed in duplicate and repeated twice.

### 2.6. Limit of Detection of SYTO-LAMP, SYBR Green-LAMP, and Neutral Red-LAMP

To determine the detection limit of the LAMP assays in this study, the DNA from *P. knowlesi* strain A1H1 culture was extracted by using the alternative DNA extraction method. A 10-fold serial dilution of the stock (1 to 0.000001% parasitemia) was performed. Four microliters of each of the diluted DNA were used as the template. The DNA template from each of the serial dilutions was tested in duplicate and repeated twice to ensure the accuracy of the result.

### 2.7. Clinical Sensitivity and Specificity

By using nested PCR results as a reference, 95% Confidence Intervals (95% CI) for both clinical sensitivity and specificity were calculated using MEDCALC^®^ software Version 22.007 vailable at https://www.medcalc.org/calc/diagnostic_test.php (accessed on 3 March 2023).

## 3. Results

After numerous trials of optimization, the optimum concentration of SYTO-9, SYBR green, and neutral red dyes tested for each LAMP assay was 50 µM, 1:400, and 100 µM, respectively.

**Figure 1:**
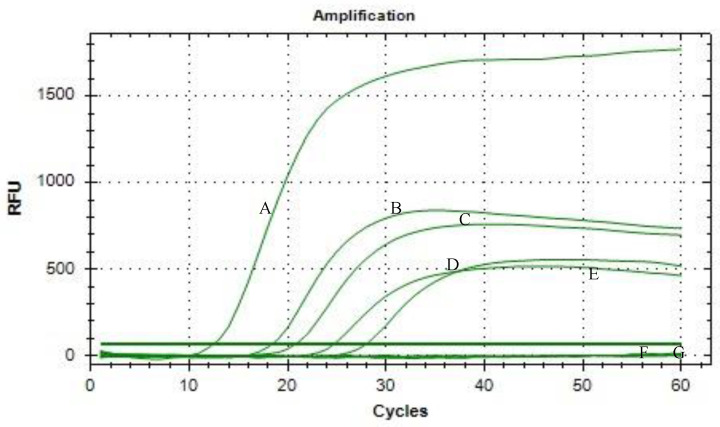
Limit of detection of SYTO-LAMP assay. A: 0.1 %; B: 0.01%; C: 0.001%; D: 0.0001%; E: 0.00001%; F: 0.000001%; G: negative control (distilled water).

**Figure 2:**
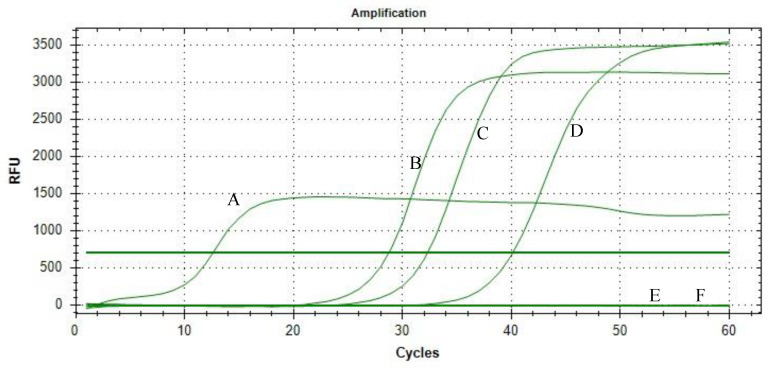
Limit of detection of SYBR green-LAMP assay. A: 0.1 %; B: 0.01%; C: 0.001%; D: 0.0001%; E: 0.00001%; F: negative control (distilled water).

**Figure 3:**
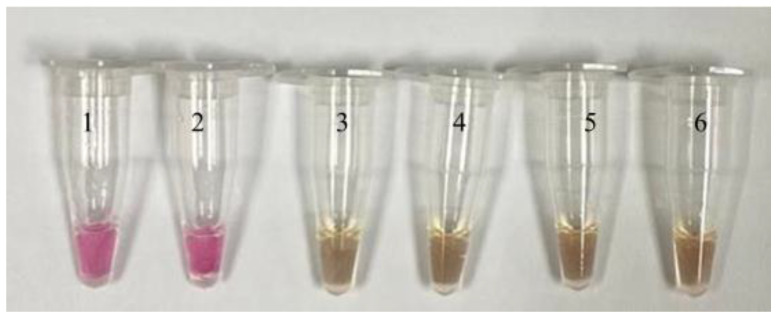
Limit of detection of the neutral red-LAMP assay. For the positive reaction, the color of the end products shifted from colorless to pink, while for the negative reaction, the color of the end products shifted from colorless to orange. Tube 1: 0.1%; tube 2: 0.01%; tube 3: 0.001%; tube 4: 0.0001%; tube 5: 0.00001%; tube 6: negative control (distilled water).

Figure 1, Figure 2 and Figure 3 show the results of the limit of detection for both SYTO-LAMP and SYBR green-LAMP and neutral red LAMP assays. Figure 3 shows the positive and negative reactions of the neutral red-LAMP assay. For the positive reaction, the reaction shifted from colorless to pink, while the negative reaction shifted from colorless to orange. By using nested PCR as the reference method, both SYTO-LAMP and SYBR green-LAMP had a sensitivity of 100% (95% CI: 94.6–100%) and 100% specificity (95% CI: 88.4–100%) while neutral red-LAMP had a sensitivity of 90% (95% CI: 79.4–95.6%) and 100% specificity (95% CI: 88.4–100%). The limit of detection of SYTO-LAMP, SYBR Green-LAMP, and neutral red-LAMP was 0.00001%, 0.0001%, and 0.01%, respectively.

Clinical sensitivity and specificity for each of the LAMP assays were shown in Table 2. Out of 67 *P. knowlesi* positive samples, neutral red-LAMP was not able to detect six samples with parasitemia below 0.01%. Meanwhile, both SYTO-LAMP and SYBR green LAMP assays detected all *P. knowlesi* positive samples without cross-reactivity with non-*P. knowlesi* samples.

## 4. Discussion

DNA extraction is the first and critical step in any disease diagnosis test. Direct DNA amplification from whole blood samples is challenging due to the interference of hemoglobin. Typically, commercial DNA extraction kits are the first of choice for this purpose. These standard protocols may involve multiple steps of purification and elution. Overall DNA extraction using these protocols takes approximately ~50 min to complete. To save time and cost, many alternatives have been explored for DNA extraction from whole blood. Such methods include chemically treated samples with Tris-EDTA buffer and proteinase K [31] and Chelex-100 chelating resin [32]. Both methods require at least two rounds of incubation at different temperatures and a centrifuge. In 2017, Piera et al. demonstrated a ‘boil and spin’ method in combination with an Eiken LAMP kit during the detection of *P. knowlesi*, *P. falciparum*, and *P. vivax* in co-endemic areas in Malaysia. However, this commercial LAMP kit (USD 1.06/reaction) is 2× more costly compared to our simple extraction method combined with LAMP (USD 0.58/reaction) [33]. Port et al. reported a microwave extraction method for *P. falciparum* from whole blood samples. Although this is a simple-to-use method, it possesses a high risk of breakage of the tubes and subsequently leads to contamination hazards. Moreover, the minimum size of the tube should not be below a capacity of 500 µL [34].

In this study, we developed a simple DNA extraction protocol for malaria blood samples. We focused on *P. knowlesi* detection as this parasite is responsible for the major malaria infections in Malaysia among the five species of human *Plasmodium* species [1,2]. The extracted DNA could be employed directly as a template without the need for further purification steps. As compared to the commercial DNA extraction kit (USD 4.97/reaction), the new DNA extraction protocol developed here offers a simple and low-cost (USD 0.11/reaction) alternative for DNA extraction from whole blood in laboratories with limited resources. Moreover, the new extraction protocols took only 5 min to complete as compared to the commercial DNA extraction protocol (~50 min).

We achieved 100.0% and 91.0% sensitivity for fluorescent LAMP-based (SYTO-LAMP and SYBR green-LAMP) and colorimetric LAMP-based assays (neutral red-LAMP), respectively, by using heat lysis coupled with dilution. As compared to other published reports, our study is superior as the results of SYTO LAMP-based here show a lower detection limit of 0.00001%, which is approximately equivalent to 0.5 P/µL. Lucchi et al. detected *P. falciparum* directly from heat-treated blood samples and managed to detect down to 4 P/µL [35]. Moreover, Modak et al. managed to detect *P. falciparum* directly from heat lysis coupled with dilution down to 1.5 P/µL [26].

LAMP has been increasingly used as new a diagnostic for infectious diseases due to its advantages over traditional PCR. Our results show that fluorescent LAMP-based assays have a better performance than colorimetric-based LAMP assays. Colorimetric-based LAMP was not able to detect six low parasitemia samples. Moreover, we observed that the color change of the end products was not significant. The weak result of colorimetric LAMP may be due to low levels of by-products such as pyrophosphate and protons. Colorimetric-based LAMP detects turbidity that was triggered by the accumulation of magnesium pyrophosphate, or color changes due to the incorporation of pH-sensitive dyes and DNA-intercalating dyes into the reaction [36]. To better discriminate between positive and negative reactions, it was recommended that placing the tubes on ice immediately after the LAMP reaction may enhance the color formation [37]. On the other hand, both fluorescent-based LAMP assays successfully detected all *P. knowlesi* samples. The result could be read directly from the amplification peak. To minimize false-positive results, a melt curve was included in each reaction. An absence of a double peak meant the absence of primer-dimer formation during the reaction.

Although the sensitivity detection level of both fluorescent-based LAMP assays was lower than that of the colorimetric-based LAMP assay, there were still some limitations to both fluorescent-based assays. They require a real-time PCR machine to conduct the test. This may hinder the use of fluorescent-based LAMP assays in resource-constrained areas. In contrast, the colorimetric-based LAMP detection assay only requires a mini heating block for amplification, this technique can be deployed as an alternative LAMP detection method in locations with limited laboratory infrastructure.

Several fluorescent-based LAMP assays have been reported to date. Quyen et al. reported that SYTO 9, SYTO 82, SYTO 16, SYTO 13, and Miami yellow were suitable to use for closed-tube real-time fluorescence detection with minimal amplification inhibition [38]. Here, we tested the efficiency of the simple extraction approach using SYBR green I and SYTO-9. We observed that the amplification speed of SYBR green-LAMP was approximately 6 min slower than SYTO-LAMP. It might be because of the inhibitory effects of SYBR green on *Bst* DNA polymerization [39,40]. This result suggests that SYTO-9 is a better fluorescent dye of choice compared to SYBR green for LAMP assays.

To minimize the inhibitory effects of fluorescent DNA intercalating dyes on the reaction, the working stock of both SYTO-9 and SYBR green was diluted with distilled water instead of dimethyl sulfoxide (DMSO). We observed that a high amount of both SYTO-9 (more than 50 µM) and SYBR green (more than 1:400) was not suitable to use for LAMP monitoring. Similar findings were found from those reported by Gudnason et al. and Eischeid [39,40]. Therefore, only a small amount of SYTO-9 and SYBR green was enough for amplification, subsequently helping to minimize the cost per reaction.

The fluorescent SYBR green-LAMP assay here showed a better performance compared to our previous report on the diagnosis of knowlesi malaria [41]. We achieved 100.0% sensitivity in the current study as compared to the previously reported (97.1%). From the previous report, the LAMP results were evaluated based on the color change in SYBR green of the end products. Upon the completion of amplification, a positive reaction is indicated by a change from orange to green. However, the color change in SYBR green was sometimes not significant for low parasitemia samples. Here, we noticed that samples of low parasitemia could be detected effectively by an RT-PCR machine compared to observing the color change of the end products as previously reported.

Over the past decades, the inclusion of fluorescent dyes in LAMP for the detection of human *Plasmodium* spp. (except for *P. knowlesi*) has been described. In 2010, Lucchi et al. described a RealAmp method for the detection of *P. falciparum* by using SYBR green [35]. Patel et al. reported a LAMP assay for the detection of *P. vivax* by using SYTO-9 and SYBR green and found that both dyes did not show any inhibitory effects on the reaction [42]. To the best of our knowledge, this is the first report on the detection of *P. knowlesi* using SYTO-9 in LAMP.

In general, there are shortcomings with LAMP. Carryover contamination is a common issue due to the high sensitivity nature of LAMP, resulting in the misreading of the results. This challenge can be easily eliminated by careful pipetting, and having a partition of the rooms or bench according to functions, such as DNA extraction, LAMP master mix preparation, and DNA template addition, to avoid frequent exposure of the tube to the surroundings. To avoid aerosol pollution, we overlaid a thin layer of mineral oil as a sealant on the liquid surface of the reaction tubes.

## 5. Conclusions

We presented a simple and purification-free extraction method for *P. knowlesi* whole blood samples. The extracted DNA could be amplified successfully by both fluorescent LAMP-based assays and colorimetric LAMP-based assays. The colorimetric LAMP-based assay shows a slightly lower sensitivity than both fluorescent LAMP-based assays. Combined with the simple DNA extraction approach developed here, the SYTO-LAMP assay may be employed as an alternative diagnostic tool for the real-time monitoring and detection of *P. knowlesi*.

## Figures and Tables

**Table 1:** *P. knowlesi* LAMP primers used in this study.

	Sequence (5′ to 3′)
FIP	GTTGTTGCCTTAAACTTCCTTGTGTTCTTGATTGTAAAGCTTCTTAGAGG
BIP	TGATGTCCTTAGATGAACTAGGCTTTGCAAGCAGCTAAAATCGT
FLP	TAGACACACATCGTT
BLP	GCACGCGTGCTACACT
F3	CCATCTATTTCTTTTTTGCGTATG
B3	CAGTGGAGGAAAAGTACGAA

FIP: forward inner primer; BIP: backward inner primer; FLP: forward loop primer; BLP: backward loop primer; F3: forward primer; B3: backward primer.

**Table 2:** Limit of detection, clinical sensitivity, and specificity of SYTO-LAMP, SYBR Green-LAMP, and Neutral red-LAMP.

	SYTO-LAMP	SYBR Green-LAMP	Neutral Red-LAMP
Limit of detection (parasitemia)	0.00001%	0.0001%	0.01%
Clinical sensitivity	100% (95% CI: 94.6–100%)	100% (95% CI: 94.6–100%)	91% (95% CI: 81.5–96.6)
Clinical specificity	100% (95% CI: 88.4–100%)	100% (95% CI: 88.4–100%)	100% (95% CI: 88.4–100%)

## Data Availability

The authors can confirm that all relevant data are included in the article.
